# The reliability of evaporation ponds as a final basin for industrial effluent: Demonstration of an environmental risk management methodology

**DOI:** 10.1016/j.mex.2023.102055

**Published:** 2023-02-03

**Authors:** Abdeljalil Adam, Nabil Saffaj, Rachid Mamouni

**Affiliations:** Laboratory of Biotechnology, Materials, and Environment, Faculty of Sciences, University IBN ZOHR, 80000 Agadir, Morocco

**Keywords:** Environment, Industrial effluents, Risk management, Evaporation ponds, Wastewater Risk management using ALARP criteria

## Abstract

Recognizing and assessing environmental risk are key components of every industry management strategy. Projects need to make sure that a detailed environmental risk management strategy is applied by methodically recognizing and addressing threats from internal and external influences to comply with regulatory standards for environmental preservation and safeguarding. This study's goal is to use a novel technique to assess the impact of environmental risks related to the use of evaporation ponds as final basins for industrial effluents. It employs qualitative and statistical methodologies to identify areas where engineering and managerial safeguards' structure, functioning, and lines of defense have flaws that might result in an ecologically hazardous occurrence. Additionally, it will offer a risk evaluation based on the gravity of the impact and the likelihood that the environmental occurrence would happen by using evaporation ponds to store industrial effluents. While the environmental threat would be entirely removed, it must be capable of reducing it to ALARP. The environmental risk assessment matrix will serve as a key factor in determining whether the environmental risk level linked with an evaporation pond is acceptable, as determined by the likelihood and impacts. The result of this research allows industrial units to recognize and control potential environmental risks associated with effluents by practically implementing a new environmental risk matrix based on several environmental and ecological effects with probability factors.

• This study aims to assist industrial operators, especially power plants, manage environmental risk by combining ALARP concepts with other factors to evaluate risk acceptance and tolerance levels.
• The Physico-chemical characteristics of effluent collected in the evaporation pond reveal that evaporation has a deleterious impact on such industrial effluent, as evidenced by a large increase in various effluent properties, some of which exceed the limit values.
• A risk evaluation found that effluent collected in the evaporation pond has a detrimental negative impact on industrial effluents. This was evidenced by a significant rise in associated activities. This could increase the expense of operating and managing evaporation ponds, which could harm the ecosystem.

## Graphical abstract

**Figure:**
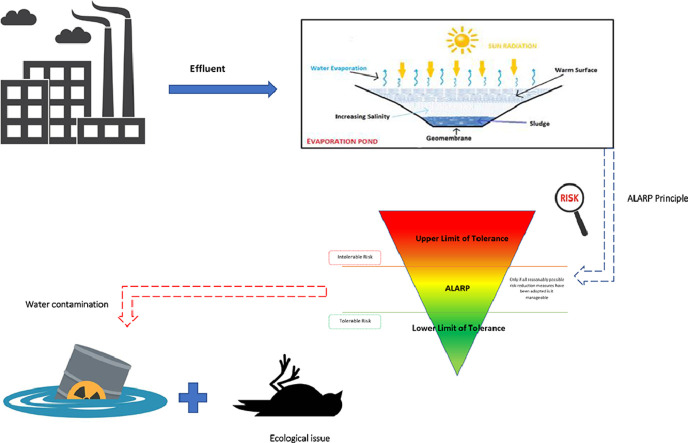

Specification table

**Table:** 

Subject area:	Environmental Science
More specific subject area:	Water Resources
Name of your method:	Wastewater Risk management using ALARP criteria
Name and reference of original method:	NA
Availability Source:	NA

## Introduction

Because of their harmful and long-lasting effects on the environment, toxic substances and other hazardous chemicals continue to be a significant class of environmental contaminants. One of the most hotly contested issues regionally, locally, and internationally is environmental deterioration, which has captured the interest of the whole globe [9].

The probability of a bad situation, the future potential occurrence of negative effects arising from an incident, the gathering of types of damage from the operation and likely implications, or, additionally, the deviation from a final set and the associated uncertain factors are all examples of risk [1].

For such businesses, the environment in which an organization operates spans from within the organization to the entire world and encompasses the air, sea, soil, resources, plants, animals, and people, along with their adjacent interacting environments [12].

*Although they are continual steps that happen across the risk management system, surveillance, engagement, and transparency are crucial risk management concepts* [15] *.*

Finding environmental hazards Risk evaluation is an important tactic for recognizing and describing environmental threats based on their likelihood, regularity, and intensity, as well as evaluating their adverse effects such as potential financial loss, damage to one's image, and business disruption. Environmental considerations must be taken into account during the course of business operations to increase the industry's efficacy in preserving the environment [19]. Risk criteria were used to conduct risk assessments. The sector must recognize risks, evaluate the dangers they pose, and accept constant levels [2].

The objective of an evaluation of environmental risk is to identify the recognized environmental threats that are substantial. In several instances, sound judgement may play a crucial part in establishing how to handle the importance, and interaction with interested parties can aid in this regard. By establishing the extent of control through every environmental element and the intensity of the environmental effect associated with each feature, importance is identified [7].

An evaporation pond is defined as a walled earthen pond in which the concentration evaporates spontaneously due to solar radiation. As the fresh water in the ponds evaporates, the chemicals in the concentrates crystallize into the brine, which is regularly collected and thrown off-site [5].

Evaporation ponds are a remedy for industrial effluent that is fed into massive ponds and evaporated gradually using direct sunlight [18]. In several nations across the globe, they are a common method of salt water treatment [1].

In contrast, risk assessment is not typically viewed as a separate field of study. Risk concerns are addressed by integrating knowledge from finance, psychology, and technology, as well as other sciences and domains, with risk assessment concepts and procedu res [6].

*This research utilized the ideas of managing risk, identifying and assessing using the ALARP Method, and finally, the new environmental risk matrix developed by* [1]

This article's primary purpose is to deploy a novel approach employing a risk matrix [1] to estimate the environmental significance of the evaporation pond solution's hazard state. Using various methods and techniques, it identifies weaknesses in the structure, operations, and barriers of defense. offered by technical and administrative safeguards, that cause environmental non-compliance. It also includes a risk assessment depending on the severity of the impact and the probability of the environmental incident happening.

Regardless of these advantages, evaporation ponds may provide several ecological and environmental issues [1], [3]. The effluent spilled from evaporation ponds, for instance, could have a devastating effect on the ecology (soil, and groundwater contamination). In addition, since evaporation ponds are open water fields, they attract wildlife, possibly increasing the death rate of these creatures if the purity of the effluent collected in the ponds is poor and exceeds the allowed limits.

In consequence, incidents might pose an environmental concern. Nevertheless, this research concentrates on the environmental risk associated with wastewater discharges into evaporation ponds. To prevent unfavorable environmental outcomes, we must answer three crucial queries: What particular environmental dangers can evaporation ponds pose? Is there a comprehensive methodology available for assessing the environmental risk posed by evaporation ponds? Do we require fresh environmental impact analysis techniques?

*By applying the environmental risk method to an industrial plant in Morocco, we will show the problems that all industries had when they used to dump their waste into evaporation ponds.*

## Materials and methods

### The study area & risk committee

*The environmental risk approach was applied to a power plant with an integrated water treatment unit in Morocco's eastern region. This plant utilizes evaporation ponds for wastewater collection.*

*In addition to the authors, the operation manager, maintenance manager, senior plant chemist, and health safety and environment managers contributed to this practical risk analysis exercise.*

### Effluent characterization methods

*The sample was taken in July 2022, and the results were gathered using a Sigma SD900 Mobile Tester.*

*Following the standard protocol, physical-chemical and bacterial studies of the effluent were executed at the Laboratory of Biotechnology, Materials, and Environment.*

*Because there were oil remains in the wastewater from the power plant, samples were taken in specific containers (glass).*

*The appropriate techniques for the analysis of effluent* [10] *were adhered to using the Basic Protocols for the testing of Effluents.*

*Using the techniques detailed in*Table 1*, the following physical, chemical, and bacteriological analyses were performed* [4,17]*:*

**Table 1:** The analysis method used for effluents.

**PARAMETERS**	**Methods**
Thermotolerant coliforms at 44°C	NM ISO 9308–1 2007
coliforms at 36 °C	NM ISO 9308–2 2007
Escherichia coli 44 °C	NM ISO 9308–1 2007
Viable micro-organisms at 22 °C	NM ISO 6222 2007
Viable micro-organisms at 36 °C	NM ISO 6222 2007
Salmonella SPP	NF ISO 19,250
clostridia at 37 °C	NM 03.7.051 1996
Vibrio cholera	NM 03.7.051 1996
Yeasts and molds	NM ISO 9308–1 2007
pH	NM ISO 10,523–2012
Temperature	Probe thermometer
Electrical conductivity	NM ISO 7888–2001
Total dissolved salts	NM 03.7.019–1991
Total suspended solids	NM EN 872:2013
DBO5	NM ISO 5815–1 ET 2–2012
DCO	MA.315-DCO 1.1–2016
Total nitrogen	NM ISO 5664–1999
Bicarbonates	NM ISO 9963–1–2001
Nitrates	calculation
total phosphorus	NM ISO 11,885–2014
Zinc	
Iron	
Copper	
Manganese	
Cadmium	
Arsenic	
Nickel	
Lead	
Total chromium	
Cobalt	
Sodium	
Tin	
Antimony	
selenium	
aluminum	
Boron	
Silver	
Sulfates	
Mercury	NM EN 1483–2012
Cationic detergents	NM ISO 7875–1 1999
Anionic detergents	
Sulfide	Colorimetry
Cyanide	
Fluorides	
Hexavalent Chromium	NM EN 1483–2012
Phenol index	Colorimetry
Active chlorine	NM ISO 11,885–2014
chlorine dioxide	NM ISO 11,885–2014
AOX	NM ISO 6107-2
Hydrocarbons	NM 00.6.151
Oils and fats	NM ISO 872 (2013)

### Risk management principles

The objective of risk management is to protect individuals, the ecosystem, and resources against the detrimental effects of human activities and natural catastrophes. In other ways, risk management enables the industry to design methods for managing or minimizing the probable destruction caused by an activity's dangers or threats. Consequently, risk management is a collection of approaches for detecting and analysing risks; this process may also comprise mitigation strategies connected to the efficiency of every sector. [8,14].

**Fig. 1:**
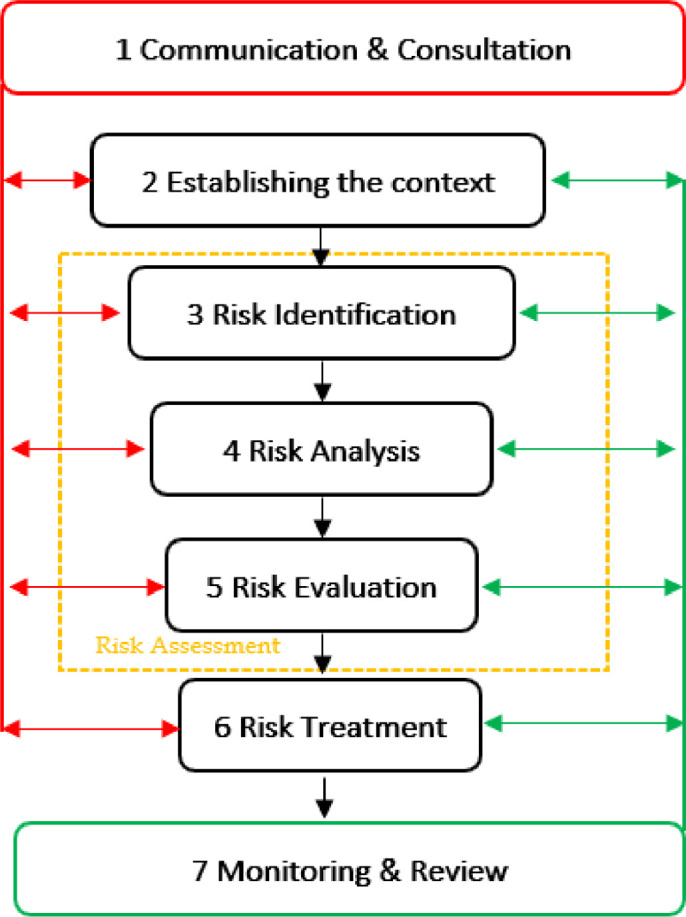
Risk management process.

*The risk management framework incorporates the following seven fundamental stages (* Fig. 1 *):*

### ALARP approach

**Fig. 2:**
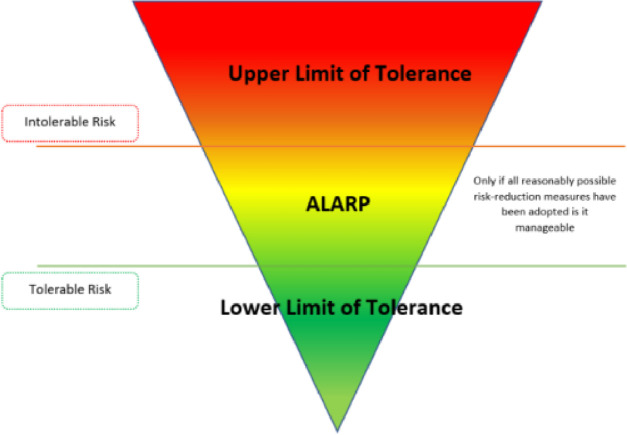
Illustration of ALARP Principle.

The American Library Association created the ALARP (Fig. 2) concept as a set of rules. Risks should be reduced to the greatest degree that is practically possible. In a different sense, all remedial measures must be taken until the expenses outweigh the potential advantages. In the framework of ALARP, thresholds for "appropriate" and "acceptable" dangers are specified [11]. (Guidance on the Demonstration of ALARP (As Low As Reasonably Practicable), 2020)

### Risk matrice

*The risk matrice implemented in this exercise is shown in* Table 2 [1]

**Table 2:** Environmental Risk Matrix (6 × 6).

*This matrix provides clear knowledge of the risk priority and the associated measures for every scoring group, as shown in* Table 3 *:*

**Table 3:** Environmental Risk priority and controls.

*Where the scores are described in* Tables 4 *and* 5 *.*

**Table 4:** Environmental consequences.

**Score**	**Environmental**
**A**	Quickly controlled, small leaks or spills (technology use) There is no moral impact
**B**	leakage or spillage of harmful and dangerous materials in a closed space. A negligible immediate influence on the neighborhood's standing.
**C**	Dangerous substances that leak or spill can be handled internally and don't need to be reported to other parties. A relatively brief effect on the local society's image
**D**	Spills and leaks of contaminants that need to be reported externally, but can still be controlled by the inside world. A temporary negative effect on the country's image
**E**	In the case of a spill or leak involving dangerous materials, external notification and the activation of external assistance are necessary. Serious harm to another's image abroad
**F**	If a large amount of a dangerous chemical leaks or spills, the government may have to take action. Financial and reputational harm on a global scale

**Table 5:** Likelihood criteria.

**Likelihood**	**Score**	**Criteria**
**Not Possible**	01	Never, ever occurred.
Very Unlikely	02	Never has this occurred in a related sector but in a distinct production process.
Unlikely	03	Occurred once every 10 years within the same business;
Possible	04	Occurred once every 05 years within the same business;
Likely	05	Occurred once every 01 years within the same business;
Very likely	06	Occurred several times during the year.

### Risk velocity and risk direction

• In risk assessments, “risk velocity” is a criterion used in combination with risk impact and likelihood.
• Velocity is a gage of how soon an anticipated risk's effects may materialize once the risk event has taken place (Time to Impact approach).
• The velocity evaluation can help choose monitoring and mitigation strategies that take into account how quickly risks show their effects. Eventually, it will give a second way to rank risks besides the total risk score.

**Fig. 3:**
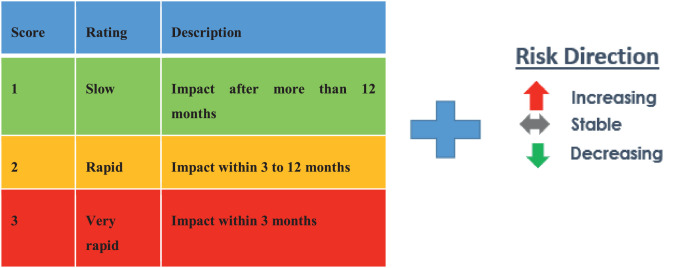
Velocity and Risk Direction.

The below scale could be used to assess velocity (Fig. 3) :

This scale, which may be changed if it turns out to be irrelevant, will be used to evaluate the risk velocity.

Ø **Internal Control Effectiveness Assessment (Illustration)**

Table 6 *describes the scoring for each Control Effectiveness Assessment.*

**Table 6:** Control effectiveness scoring.


**Score**	**Descriptions - the extent to which current controls reduce risk**
**1**	The design, implementation, and operation of controls are efficient. With the risk score reduced to a minimally acceptable level, no more mitigations are necessary to support the achievement of the objectives. Concerning the effectiveness of controls in lowering risk impact and likelihood, all stakeholders agreed. Assignment of management responsibility and thorough documentation of controls.
**2**	controls that are functional and well-designed. In light of recent changes to operations, it is important to establish that accountabilities are in existence, understood, and actively handled.All stakeholders have recorded the controls and rated the risk as acceptable, requiring no additional or minimal mitigations. Alternatively, due to the nature of the risk, measures cannot be more effective than adequate.
**3**	Minor weaknesses and improvement possibilities have been identified, and action plans will be put into place. The controls that are already in place don't manage the risk well enough, and there are some actions and procedures that could further lower the risk.
**4**	Current measures are either in the process of being adopted or are ineffective. To further control the risk, new mitigations might be implemented. There is no defined timetable or clear accountability for work completion.
**5**	High-risk exposure if existing measures are insufficient. There is no responsibility assigned for the little controls in place, and the risk score is high or the trend is increasing. To lower the risk to the level that management expects, significant steps are needed. It is necessary to describe existing controls and new mitigations, assign owners, and monitor their applications.
**6**	controls that are ineffective or do not function as intended. Stakeholders have determined that the risk score is at an unacceptable level, and new mitigations, procedures, and systems are needed to lessen the effect and probability of the risk. The new mitigations must be documented, implemented, and monitored. Management and the Board may also need to be notified.
**7**	There is no strategy, action plan, or structure in place to control the risk. The organization is immediately exposed to the risk's full impact, and other methods to lessen that impact have not been found. Management and the Board must recognize the effect and provide the risk owners with instructions to reduce the risk to an ALARP level.

## Results and discussion

### Characterization of effluents stored in evaporation ponds

The following parameters illustrated in Table 7 surpass the permissible values established by Moroccan rules for effluents discharged into superficial or subsurface waterways (Moroccan limit values of discharges, 2017) [16] according to the results of the effluent Physico-chemical study of the evaporation pond:

• *The electrical conductivity is 7.85 mS/cm, which is greater than the limit value of 2.7 mS/cm.*
• *The DBO5, with a value of 253* *mg O2 l, exceeds the limit value of 100* *mg O2/l.*
• *The DCO, with a value of 625* *mg O2/l, exceeds the limit value of 500* *mg O2/l.*
• *The sulfates, with a value of 1563* *mg /l, exceed the limit value of 600* *mg/l.*
• *The Anionic detergents with a value of 3.36* *mg /l exceed the limit value of 3* *mg/l.*
• *With a value of 3.1* *mg/l, the phenol index exceeds the limit value of 0.5* *mg/l.*
• *The active chlorine value of 0.5* *mg Cl2/l is greater than the limit value of 0.2* *mg Cl2/l.*
• *The chlorine dioxide concentration of 0.1* *mg / l, exceeds the limit value of 0.05* *mg/l.*

**Table 7:** Effluents characterization results.

PARAMETERS	Units	Results	Limit values	Conformity
Thermotolerant coliforms at 44 °C	UFC / 100ml	<3	Absence	Not Compliant
coliforms at 36 °C	UFC / 100ml	Not detected	Absence	Compliant
E. coli 44 °C	UFC / 100ml	<1	Absence	Not Compliant
Viable micro-organisms at 22 °C	UFC / ml	11	Absence	Not Compliant
Viable micro-organisms at 36 °C	UFC / ml	<1	Absence	Not Compliant
Salmonella SPP	Dans 1 L	Not detected	Absence	Compliant
clostridia at 37 °C	UFC / 100ml	Not detected	Absence	Compliant
Vibrio cholera	Dans 5000ml-[1]/Dans 450ml-[2]	Not detected	Absence	Compliant
Yeasts and molds	UFC / ml	1.2	Absence	Compliant
pH	–	7.75	5.5–9.5	Compliant
T emperature	°C	25.3	30	Compliant
Electrical conductivity	ms/cm	7.85	2.7	Not Compliant
Total dissolved salts	g/l	5.26	–	Compliant
Total suspended solids	mg/l	21.3	100	Compliant
DBO5	mg O2/ l	253	100	Not Compliant
DCO	mg O2/ l	625	500	Not Compliant
Total nitrogen	mg/l	12.6	40	Compliant
Bicarbonates	mg/l	356.3	–	Compliant
Nitrates	mg/l	1.86	-	Compliant
total phosphorus	mg/l	2.36	15	Compliant
Zinc	mg/l	1.12	5	Compliant
Iron	mg/l	0.12	5	Compliant
Copper	mg/l	0.15	2	Compliant
Manganese	mg/l	0.15	2	Compliant
Cadmium	µg/l	0.2	250	Compliant
Arsenic	µg/l	23	100	Compliant
Nickel	µg/l	5.2	5000	Compliant
Lead	µg/l	4.6	1000	Compliant
Total chromium	µg/l	4.8	2000	Compliant
Cobalt	µg/l	5.1	500	Compliant
Sodium	mg/l	785	–	–
Tin	µg/l	4.69	2500	Compliant
Antimony	µg/l	4.89	300	Compliant
selenium	µg/l	5.15	100	compliant
aluminum	µg/l	6.12	10,000	compliant
Boron	mg/l	0.86	–	Compliant
Silver	µg/l	63.2	100	Compliant
Sulfates	mg/l	1563	600	Not Compliant
Mercury	µg/l	0.15	50	Compliant
Cationic detergents	mg/l	1.02	3	Compliant
Anionic detergents	mg/l	3.36	3	Not Compliant
Sulfide	mg/l	<0.1	–	Compliant
Cyanide	mg/l	0.01	0.5	Compliant
Fluorides	mg/l	2.13	20	Compliant
Hexavalent Chromium	mg/l	0.01	0.2	Compliant
Phenol index	mg/l	3.1	0.5	Not Compliant
Active chlorine	mg Cl2/l	0.5	0.2	Not Compliant
chlorine dioxide	mg/l	0.1	0.05	Not Compliant
AOX	µg/l	230	5000	Compliant
Hydrocarbons	mg/l	4	–	–
Oils and fats	mg/l	1.3	30	Compliant

The toxicity of this wastewater released into the evaporation pond might be the cause of the existence of four bacteria (Thermotolerant coliforms, Escherichia coli, and Viable micro-organisms at 22 °C and 36 °C) in the effluent [13].

Microorganisms are the most diverse group of pathogenic strains found in wastewater. The human digestive system is home to a wide variety of microorganisms, most of which are expelled in feces. The preponderance of bacterial infections in the effluent are foodborne bacterial diseases, even though many of these microorganisms are benign and helpful to their hosts.

*Specifically, when releases enter outdoor recreation waterways or during recycling and treatment, effluent poses a serious health risk by acting as a germ storage space.*

The results of analyzing the Physico-chemical characteristics of the effluent dumped into the evaporation pond demonstrate that evaporation negatively influences such effluents, as shown by a notable rise in various effluent characteristics, some of which exceed the limit values. This consequence might have detrimental effects on the ecosystem and ecology. Hence, we examine a rise in water contamination that leads to serious environmental threats.

### Benchmark survey

**Fig. 4:**
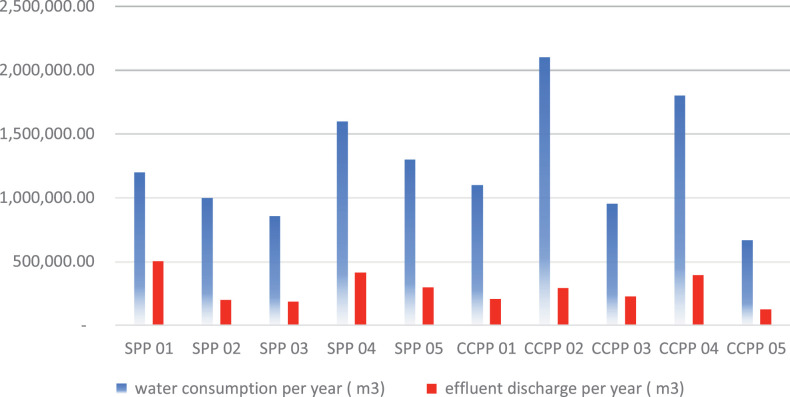
The annual water consumption vs the annual effluent discharged into the evaporation pond.

A global survey of ten electricity production sites that use evaporation ponds for their effluent was conducted to determine the amount of industrial effluents discharged into evaporation ponds as the ultimate basins for the effluent end-life utilised by industries. The results of this survey are presented in Fig. 4 and Table. 8.

**Table 8:** The Benchmark survey result.

	water consumption per year (m3)	effluent discharge per year (m3)	Effluent discharged per**%**
**solar power plant 01**	1200,000.00	504,000.00	18
**solar power plant 02**	1000,000.00	200,000.00	20
**solar power plant 03**	860,000.00	189,200.00	22
**solar power plant 04**	1600,000.00	416,000.00	26
**solar power plant 05**	1300,000.00	299,000.00	23
**combined-cycle power plant 01**	1100,000.00	209,000.00	19
**combined-cycle power plant 02**	2100,000.00	294,000.00	14
**combined-cycle power plant 03**	952,000.00	228,480.00	24
**combined-cycle power plant 04**	1800,000.00	396,000.00	22
**combined-cycle power plant 05**	669,000.00	127,110.00	19

The results of the survey show that 21% of water is wasted on the median and either gathered in evaporation ponds or thrown outside. This proportion is considerable and could have a big impact on how resources are used in the business, especially at this period in history when there are so many persistent constraints. The most obvious manifestation is the absence of water due to a shortage of rainfall. That has significant negative effects on the environment and many important sectors. It can be difficult to evaluate these consequences generally.

*It is suggested that wastewater reuse or recycling using a range of technological techniques serve as an essential form of control to lessen potential environmental and ecological threats brought on by industries.*

### Environmental risk identification

After several meetings of the risk management committee on the use of the evaporation ponds and based on the environmental accidents that have occurred in the evaporation ponds in the past, the following major risks (Table 9) have been recognized and agreed upon by the committee.

**Table 9:** Environmental risk assessment scoring results.

*Based on the results of this practical risk analysis, the following interpretations can be brought up:*

In accordance with the environmental risk priority and control criteria, five activities related to the use of evaporation ponds—wastewater collection in the evaporation ponds and the effluents pits, in addition to the production of process water—have red color scoring (high priority and intolerable risk). These risks need to be eliminated immediately.

According to the Environmental Risk priority and controls criteria, four activities (cleaning of evaporation ponds, wastewater collection in the evaporation ponds, and the effluent pits) related to the use of evaporation ponds have yellow colors scoring (Medium to high priority + intolerable risk); those risks should be properly managed.

Using the findings of the risk analysis, it was determined that the wastewater deposited into the evaporation pond had a negative influence on such industrial effluents. This was demonstrated by a sharp increase in the number of related activities. The expense of maintaining and running the evaporation ponds might go up because of this effect, which could also have detrimental environmental and ecological effects.

### Control effectiveness and action plan

*The control effectiveness of the high environmental risks associated with the use of evaporation ponds is shown in* Table 10 *:*

**Table 10:** Environmental risk assessment’ control effectiveness, velocity, and risk direction.

The wastewater collection in evaporation ponds, where high levels of water pollutants are released with wastewater, and the cleaning of evaporation ponds, where the production of a significant amount of sludge (solid waste) is the primary environmental threat, revealed the highly ineffective control measures that are currently in place.

*Wastewater spills from evaporation ponds and ecological issues were also demonstrated to have highly inefficient control measures, which is 05, in the wastewater collected in the evaporation ponds.*

*The velocity varies between 1 for the wastewater spill from evaporation ponds and the generation of a huge amount of sludge and 2 for ecological issues and high water pollutants released with wastewater.*

For the risks that received a score of 1 (slow rating), this may be explained by the possibility that the impacts of the predicted risk could manifest after the risk event has occurred; therefore, the influence would not be felt for more than a year.

*Other risks with a score of 1 (rapid slow) may be explained by the fact that the risks' consequences may manifest 3–12 months after the risk event has occurred.*

The risk direction remains stable for high water pollutants released with wastewater, decreases for the ecological issue, and increases for both the generation of a huge amount of sludge and the wastewater spill from evaporation ponds.

**Fig. 5:**
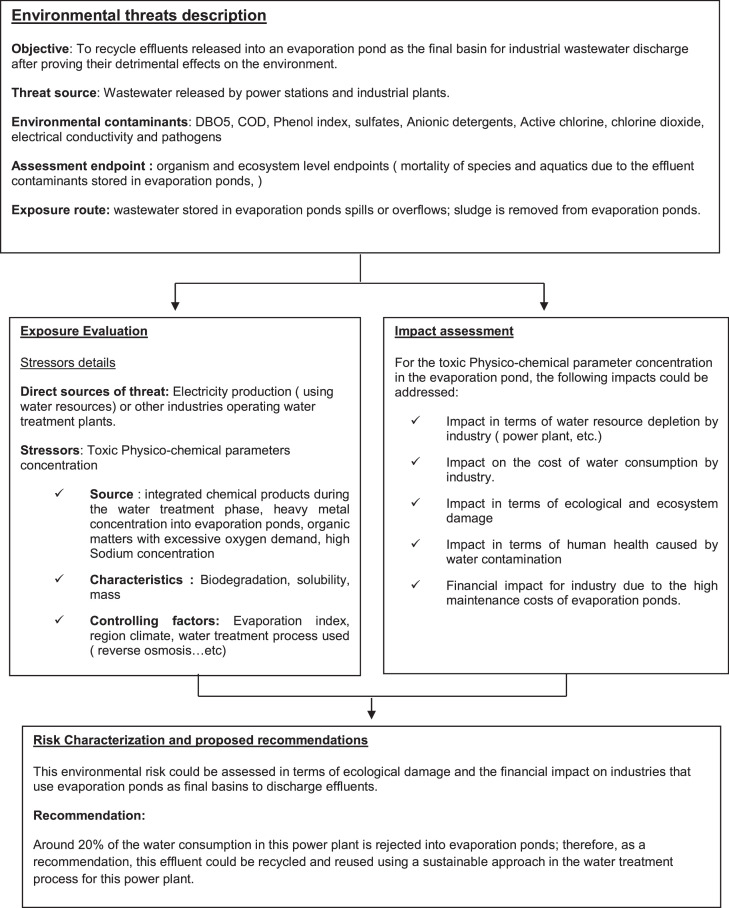
Framework for the environmental risk assessment of effluent discharged into evaporation ponds.

*The Framework for the environmental risk assessment of effluent discharged into evaporation ponds* [20] *is shown in* Fig. 5

Based on numerous benchmarks for solar power plants, they used evaporation ponds to store their wastewater. The idea behind this solution is to avoid releasing wastewater directly into the environment (via dams, sea, rivers, or directly into the atmosphere). This solution is used when comparing the results of this study with other recent studies that recommend evaporation ponds as final effluent storage and release, especially for solar power plants that use water vapor as the main input to run the steam turbine.

Future researchers could look into the possibility of directly recycling wastewater rather than dumping it into an evaporation pond. They could also investigate the possibility of using the solar collectors already in place for the process to heat this wastewater and then inject it into a solar sill to produce freshwater that could be reused by those solar power plants, thereby optimizing the consumption of fresh water to contribute to the necessary sustainability.

Because evaporation pond solutions are only implemented in dry, sunny regions, the use of the environmental risk method for an industrial plant in Morocco is only intended to serve as a case study. However, we can generalize the disadvantages of all industries around the world that may discharge their effluents into evaporation.

Placing large sustainable solar stills as a replacement for evaporation ponds is one of the eco-friendly alternatives for businesses that use evaporation ponds; the advantage is protecting the environment as well as recycling wastewater. This will enable the industry to directly reuse its wastewater with reducing water consumption costs.

## Conclusion

The purpose of this research is to present a clear method that can assist industrial owners, particularly power plants, in managing environmental risk by employing a unique technique for evaluating risk acceptance and tolerance levels that combines ALARP principles with some other elements.

The findings of assessing the Physico-chemical properties of the effluent deposited into the evaporation pond indicate that evaporation has a detrimental impact on such effluents, as evidenced by a significant increase in several effluent characteristics, some of which exceed the limit values. This conclusion may have negative ecological and economic repercussions. Consequently, we investigate the growth in water pollution, which has resulted in severe environmental hazards.

Utilizing the results of a risk assessment, it was concluded that the wastewater released into the evaporation pond had a detrimental impact on industrial effluents. This was evidenced by a significant rise in associated activity. This effect might increase the cost of maintaining and operating the evaporation ponds, which could have negative environmental and ecological implications.

*Effluent reuse or recycling using a variety of sustainable technical approaches is proposed as a preventive technique for avoiding future environmental and ecological concerns.*

## Ethics statements

*All authors declared that informed consent was obtained from participants and that the platform's data redistribution policies were complied with.*

## CRediT authorship contribution statement

**Abdeljalil Adam:** Methodology, Writing – review & editing, Conceptualization. **Nabil Saffaj:** Supervision, Validation. **Rachid Mamouni:** Resources, Writing – review & editing.

## Declaration of Competing Interest

The authors declare that they have no known competing financial interests or personal relationships that could have appeared to influence the work reported in this paper.

## Data Availability

No data was used for the research described in the article.
